# Recognising the potential of large animals for modelling neuromuscular junction physiology and disease

**DOI:** 10.1111/joa.13749

**Published:** 2022-09-02

**Authors:** Stephen D. Cahalan, Ines Boehm, Ross A. Jones, Richard J. Piercy

**Affiliations:** ^1^ Comparative Neuromuscular Diseases Laboratory, Department of Clinical Science and Services, Royal Veterinary College University of London London UK; ^2^ Edinburgh Medical School: Biomedical Sciences University of Edinburgh Edinburgh UK; ^3^ Euan MacDonald Centre for Motor Neurone Disease Research University of Edinburgh Edinburgh UK; ^4^ Biozentrum University of Basel Basel Switzerland

**Keywords:** large animals, NMDs, NMJ disorders, peripheral neuropathy

## Abstract

The aetiology and pathophysiology of many diseases of the motor unit remain poorly understood and the role of the neuromuscular junction (NMJ) in this group of disorders is particularly overlooked, especially in humans, when these diseases are comparatively rare. However, elucidating the development, function and degeneration of the NMJ is essential to uncover its contribution to neuromuscular disorders, and to explore potential therapeutic avenues to treat these devastating diseases. Until now, an understanding of the role of the NMJ in disease pathogenesis has been hindered by inherent differences between rodent and human NMJs: stark contrasts in body size and corresponding differences in associated axon length underpin some of the translational issues in animal models of neuromuscular disease. Comparative studies in large mammalian models, including examination of naturally occurring, highly prevalent animal diseases and evaluation of their treatment, might provide more relevant insights into the pathogenesis and therapy of equivalent human diseases. This review argues that large animal models offer great potential to enhance our understanding of the neuromuscular system in health and disease, and in particular, when dealing with diseases for which nerve length dependency might underly the pathogenesis.

## Graphical abstract

The role of the neuromuscular junction in neuromuscular disorders is not fully understood. Naturally occurring neuromuscular diseases in large animals might offer translational benefits over lab rodent studies.

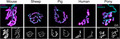

## 1 INTRODUCTION

**FIGURE 1:**
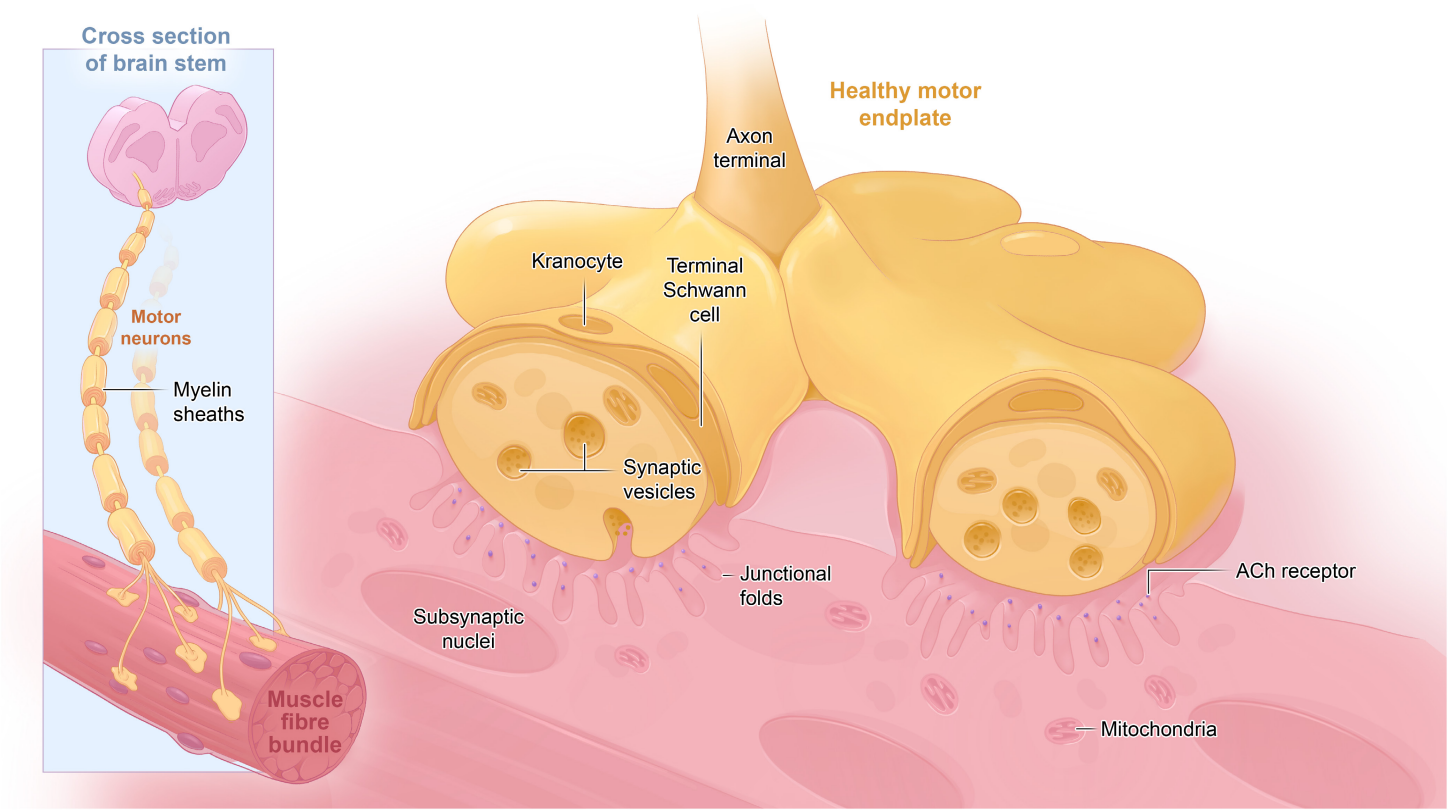
A schematic diagram of the healthy motor axon. The brainstem is shown as this houses the motor neurons for the longest motor nerves of many large mammals, including horses.

Within the neuromuscular system, the neuromuscular junction (NMJ) plays a fundamental role: this highly specialised synapse transmits signals from motor neurons (MNs) to skeletal muscles (Sanes & Lichtman, 1999). NMJs are comprised of four basic cell types: the pre‐synaptic motor neuron and its axon (which terminates in the pre‐synaptic nerve terminals); the post‐synaptic muscle fibre, which contains the post‐synaptic motor endplate; terminal Schwann cells capping the nerve terminal (Alhindi et al., 2021) and kranocytes, which cap the NMJ (Court et al., 2008). For the majority of skeletal muscles, each fibre has one NMJ (Nishimune & Shigemoto, 2018) (Figure 1—schematic healthy NMJ), but innervation patterns differ between species and muscles: the sternomastoid muscle for example can have up to seven endplate bands in the rabbit, compared to a single band in humans and mice (Paul, 2001).

**FIGURE 2:**
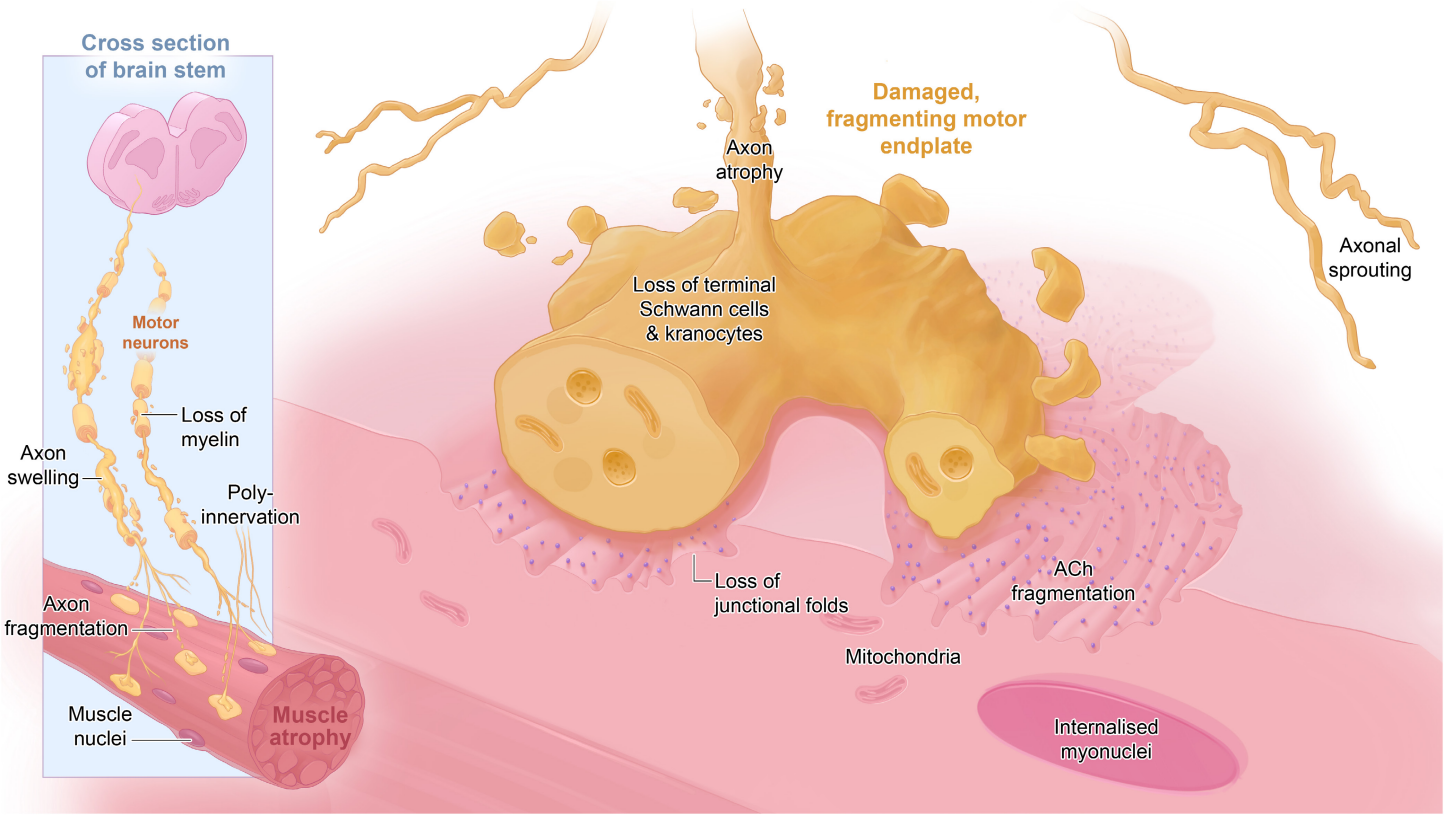
A schematic diagram of the unhealthy motor axon. The brainstem is shown as this houses the motor neurons for the longest motor nerves of many large mammals, including horses.

Recognition of the crucial role of the NMJ in the facilitation of movement sparked interest in the study of the peripheral nervous system (PNS) as early as the 1700s (Lin & McArdle, 2021). Changes in pre‐ or post‐synaptic NMJ size and/or configuration, and structural changes of the motor neuron or post‐synaptic muscle fibre, play a significant role in neuromuscular disease pathogenesis. For example fragmentation of the endplate (Slater, 2019), withdrawal of the motor nerve (denervation) (Chung et al., 2017; Sleigh et al., 2020; Wernig & Herrera, 1986), poly‐innervation and axonal sprouting and loss of/clumping of terminal neurofilaments are well‐recognised features of NMJ remodelling (Cifuentes‐Diaz, 2002; Gordon et al., 2004; Wernig & Herrera, 1986) (Figure 2—schematic of diseased NMJ). Subsequently, the identification of a structure–function relationship at the NMJ, such as myelination of the motor axon for rapid neurotransmission, active zones juxtaposing acetylcholine receptors for targeted release of synaptic vesicles containing neurotransmitter and a ‘safety factor’ guaranteeing generation of evoked end‐plate potentials, suggested that the study of NMJ morphology could teach us not only about the basic physiology of the neuromuscular system, but could also help in the development of treatments for motor dysfunction (Holz & Fisher, 1999). The scientific community has used many animal models to study the impact of pathological changes on the NMJ, but these consisted predominantly of small vertebrate models such as rodents and *D. rerio* (zebrafish), and invertebrate models such as drosophila (fruit fly) or *C. elegans* (roundworm). These are popular models due to their relatively inexpensive husbandry costs, easy maintenance, and the multitude of well validated experimental techniques that are available.

This review covers aspects of comparative NMJ research in traditional rodent models, humans and large mammals. It addresses translational issues in rodent models of the NMJ, and how genetic, morphological and physiological differences between humans and animals might impact disease phenotypes and our understanding thereof. It examines the opportunities afforded by the study of large mammalian NMJs—from understanding how NMJs normally develop in a species of similar size to humans, and how these NMJs respond to injury in naturally occurring large mammalian neuromuscular diseases (NMDs), particularly in the context of length‐dependent neuropathies.

## 2 CROSS‐SPECIES ACCESSIBILITY AND GENETIC HETEROGENEITY OF THE MAMMALIAN NMJ

Neuromuscular junctions are plastic, both in function and morphology–these adaptations are muscle activity‐driven (Deschenes et al., 1993), mediated in part by skeletal muscle‐derived molecular factors such as PGC‐1α (Arnold et al., 2014). Study of the NMJ is rendered possible due to their comparatively large size and their accessible location in the peripheral nervous system. This has led to the extensive use of the NMJ as a ‘model synapse’ in both vertebrates and invertebrates (Coers & Woolf, 1959; Slater, 2015) despite the significant differences that exist between neuromuscular and inter‐neuron synapses, such as those within the synaptic cleft and the post‐synaptic membrane (Zou & Pan, 2022); nevertheless, study of the NMJ has provided deeper insights into the function of less accessible synapses within the CNS (Lin & McArdle, 2021).

Mammalian animal models are commonly used to study both NMJ function and dysfunction. Generally, the overall body plan (Bauplan) across mammals is encoded by highly conserved structural genes that determine both inter‐ and intra‐species variation (Travillian et al., 2003). However, whilst the mammalian Bauplan is highly conserved, it is precisely those inter‐species differences that define the degree of conservation; in relation to the PNS, or the NMJ in particular, this degree of cross‐species conservation and its relevance to function are less well explored. Therefore, differences between humans and other mammals must be considered carefully when translating research from animal models to humans. Since rodent models are used most commonly in biomedical research (Ellenbroek & Youn, 2016), there is a clear need to establish the similarities and differences that their NMJs share with those of humans.

## 3 TRANSLATIONAL PITFALLS IN RODENT NMJ FORM AND FUNCTION

A translation gap exists in neurophysiological and neurodegenerative disease research, driven in part by the failure of traditional laboratory models to recapitulate their human counterparts in both phenotype and pathology (Eaton & Wishart, 2017). Economic necessity, due to costs of studies in species other than traditional models, has resulted in the majority of structural and functional features of the mammalian NMJ having been historically studied using rodents (mice and rats). Beyond the obvious differences in body size between rodents and humans, and the expected variations that exist between rodent strains (Harper, 2010; Hestehave et al., 2020), there are also clear differences in NMJ form and function over the lifetime of each species that should be considered in a translational setting. First, some obvious interspecies differences exist: human NMJs are significantly smaller and more fragmented compared to their mouse counterparts, with much thinner pre‐terminal axons, more rudimentary nerve terminals and ‘nummular’ (coin‐shaped) endplates (Jones et al., 2017). Differences in neurotransmitter release represent a second distinction—the human NMJ has the smallest currently recognised nerve terminal surface area amongst vertebrates (Boehm, Alhindi, et al., 2020; Boehm, Miller, et al., 2020; Gromova & La Spada, 2020; Jones et al., 2017; Slater, 2017) and consequently, only a small quantity of the neurotransmitter acetylcholine (ACh) is released per action potential (quantal content). However, human NMJs have deeper post‐synaptic folding than mice, and the increased area containing sodium channels within the folds contributes to the amplification of the ACh signal. As such, human NMJs have a lower ‘safety factor’ (Wood & Slater, 2001) compared to those of mice (that release more ACh from larger nerve terminals). [The safety factor is a ratio that describes the capacity of neuromuscular transmission to elicit action potentials despite changes in neurotransmitter release or physiological condition. Values over 1 guarantee muscle contraction; values below 1 would indicate failure of neuromuscular transmission.] One review highlights multiple studies from various research laboratories showing that safety factors vary up to 4‐fold between muscles of a single species (Wood & Slater, 2001). Still, a comprehensive comparative analysis across multiple species and muscles is lacking. Third, when considering different species as models of NMJ disorders, NMJ stability varies over time, as does the occurrence of age‐related NMJ degeneration. Several animal ageing studies describe NMJs as inherently unstable, suggesting that motor endplates fragment as a consequence of the ageing process (Valdez et al., 2010). Until recently, it was unclear whether this was true in ageing humans (Oda, 1984) as the inherent complexity and ethical issues related to human tissue sampling have hindered further progress in this area. Interestingly, despite electrophysiological signs of unstable NMJ transmission in ageing, such as an increase in jitter and jiggle of motor unit potentials (Hourigan et al., 2015; Piasecki et al., 2016), recent work shows that NMJ morphology in select leg muscles (*extensor digitorum longus*, *peroneus brevis*, *peroneus longus* and *soleus*) is preserved across the human lifespan (Jones et al., 2017). The human NMJ also appears stable in affected muscles following traumatic injury of the brachial plexus (Gupta et al., 2020). Similarly, the NMJs of rectus abdominis appear stable in the muscle wasting associated with cancer cachexia (Boehm, Alhindi, et al., 2020; Boehm, Miller, et al., 2020), despite murine research suggesting denervation as a possible mechanism for the muscle wasting (Daou et al., 2020).

Given these differences, bridging the resulting translation gap requires more clinically relevant models of NMJ behaviour and stability that better mimic the human phenotype in health and disease, and without confounding factors such as age‐related degeneration.

The translation gap is also evident in the Charcot–Marie–Tooth (CMT) group of disorders encompassing the most common forms of human hereditary motor and sensory neuropathy (Pereira et al., 2012); the need to find appropriate models of such human diseases is especially pertinent. For example murine models carrying heterozygous mutations in the *Dynamin 2* gene, responsible for dominant‐intermediate CMT type B, do not develop signs of an axonal or demyelinating neuropathy, characteristic of the human disease (Pereira et al., 2020). Another study documented severe vocal fold paresis in humans, as a rare and sometimes life‐threatening clinical feature of CMT type 2, resulting from autosomal dominant mutations of the canonical Notch ligand Jagged1 gene (or *JAG1*). A homozygous *Jag1* mutation in mice is embryonically lethal while heterozygotes display only a mild peripheral neuropathy: focally folded myelin was the only effect noted in recurrent laryngeal nerve sections (Sullivan et al., 2020). Finally, the Yars^E196K^ mouse model of dominant intermediate CMT type C, fails to display a clear phenotype as heterozygotes; only as homozygotes do animals display very mild disease‐associated features (Hines et al., 2021).

These examples highlight the translational difficulties with some rodent disease models: clearly, researchers should be careful when extrapolating clinically relevant information, as insights into the potential phenotypic, mechanistic and therapeutic avenues can be masked by species differences. Thus, despite the historical successes of rodent models for tackling distinct research questions (e.g., elucidating the role of PGC1α in NMJ remodelling; Arnold et al., 2014), there is a need to identify models capable of more closely matching human morphology and pathophysiology.

## 4 COMPARATIVE MAMMALIAN NMJ MORPHOLOGY AND PHYSIOLOGY

As outlined in the previous section, for a model to be successful, it needs to mimic the human condition; in the context of NMJ research at least, large mammalian models might offer a solution to some phenotypic and physiological translational issues. The longer lifespan of larger mammals (for example), in comparison to rodents, has great appeal for research, as this could allow for more accurate modelling of chronic neurodegenerative disorders such as Parkinson's Disease, Spinal muscular atrophy (SMA) and Amyotrophic lateral sclerosis (ALS) (Duque et al., 2015; Eaton & Wishart, 2017; Holm et al., 2016; Yang et al., 2021) at pre‐clinical levels or for following long term treatments. For example age‐dependent changes effect readouts in ALS, and mutations in superoxide dismutase 1 (*SOD1*) are among those linked to familial forms of ALS. [In a *SOD1* ‐ *G*93A transgenic pig model, movement disorders along with *SOD1* nuclear accumulation and ubiquitinated nuclear aggregates appeared (Yang et al., 2014), something not observed in *SOD1*‐ *G*93A mouse models (Yang et al., 2021).] Thus, phenotypic differences between transgenic *SOD1* mice and pigs suggest that large animal models might recapitulate better the age‐dependent change observed in human patients.

It is important to identify larger mammalian models with NMJ morphology and physiology similar to humans, since the species differences in the functional properties of neuromuscular transmission as previously outlined could ultimately affect pre‐clinical translation. Similarity to human NMJ morphology might indicate similarity in synaptic transmission according to correlations drawn between quantal content/synaptic area and post‐synaptic folding index (Wood & Slater, 2001). Thus, similarity in overall anatomy might predict similarity in overall physiology. Therefore, future studies including in‐depth analysis of NMJ morphology via electron microscopy, combined with electrophysiological experiments, would allow measurement of post‐synaptic folds and morphometric correlation with variables of neuromuscular transmission, including quantal content and endplate potentials. Additionally, with advances in spatial transcriptomics, it is possible to link tissue morphology with its transcriptional landscape (Eng et al., 2019; Marx, 2021; Xia et al., 2019) which would facilitate correlation between NMJ morphology and sub‐cellular transcription.

The advantages and disadvantages of rodent and large animal models must be considered when studying diseases involving the NMJ. For example, the genetic pliability of rodent models helps to recapitulate the human condition in the laboratory, yet the homogeneity of their genetic background, whilst valuable in reducing the numbers of animals needed to find an effect, can hinder experimental findings–for example, about 10% of ALS patients carry familial forms of the disease, yet representative lab animal models fail to replicate the broad spectrum of human ALS phenotypes due to the greater background genetic heterogeneity within ALS patients (Picher‐Martel et al., 2016), thus affecting translational efficacy of experimental data. In contrast, many large animal models occur spontaneously on heterogeneous (outbred) backgrounds, reflecting the human condition (Casal & Haskins, 2006).

Whilst genetic conservation is one important factor (Barthélémy et al., 2019), the gross anatomy of animal models and humans should also be considered. The similarity of brain size, nerve length, muscle size, NMJ morphology and functional properties of muscles are essential factors to consider when assessing the advantages and disadvantages of animal models. One particular advantage of large animal models is the similarity in NMJ morphology to those of humans (Boehm, Alhindi, et al., 2020; Boehm, Miller, et al., 2020). Exploring this similarity could prove to be of substantial translational benefit, in particular given other anatomical similarities of the CNS; for example pig and sheep have more similar brain mass and skull thickness compared to humans, than either rodents or even non‐human primates (Pelekanos et al., 2018).

A recent study comparing selected pelvic limb muscle NMJ morphology in mouse, cat, dog, sheep, pig and human, revealed baseline data for the mammalian NMJ, laying the groundwork for subsequent comparative studies of larger mammalian NMJs (Boehm, Alhindi, et al., 2020; Boehm, Miller, et al., 2020). Whilst the study identified that sheep had the closest morphology to the human NMJ, it also concluded that there are stark differences in overall NMJ morphology between human and smaller mammalian models, that is, mouse, cat and dog. In contrast, the larger mammalian models (sheep and pig) with comparable body weight to humans, were more similar (Boehm, Alhindi, et al., 2020, Boehm, Miller, et al., 2020). For this reason, we henceforth focus on larger mammalian models–here defined as animals of a similar or larger size than humans–and the benefits that the study of their neuromuscular system could have in translational research.

**FIGURE 3:**
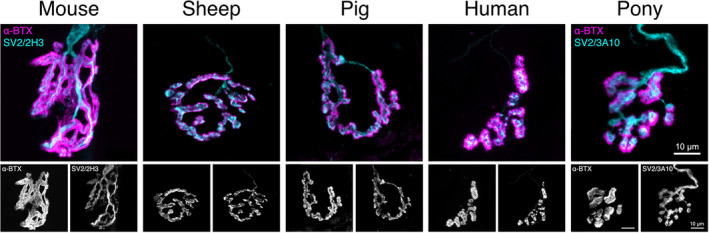
Heterogeneity of the mammalian NMJ. Confocal micrographs representing average NMJ morphology in *soleus*, a predominantly slow‐twitch pelvic/hind/lower limb muscle, across mammalian species arranged in ascending body size: the mouse, sheep, pig, human and pony. The upper panel depicts composite images of pre‐ (cyan) and post‐synapse (magenta), pseudo‐coloured in Fiji. The bottom panels depict the pre‐ and post‐synapse individually in greyscale. SV2, synaptic vesicle protein 2 (cyan); 2H3 and 3A10, neurofilament (cyan); α‐BTX (α‐bungarotoxin), acetylcholine receptors (magenta); Scale bar = 10 μm across all images.

Figure 3 illustrates the similarities in size and overall NMJ morphology between sheep, pig, pony and human and the stark contrast between NMJs in these larger mammalian models compared with those of mice. Whilst the mouse has a much larger NMJ and a wider diameter innervating motor axon, the sheep, pig and human NMJ are much more similar in overall NMJ size and axon diameter (Boehm, Alhindi, et al., 2020, Boehm, Miller, et al., 2020). Pony NMJs (Cahalan et al., 2022, under review) are strikingly similar to the human NMJ in appearance, although their terminal motor axon diameter is larger than that of humans. The suitability then of larger mammalian models as possible substitutes or valuable additions to rodent models of these neurodegenerative diseases will require further study.

## 5 UNCOVERING COMPARATIVE EVOLUTIONARY RELATIONSHIPS AT THE NMJ

Aside from linking the relationship between NMJ morphology and physiology, a better understanding of the underpinning genetic mechanisms might help address gaps in translational understanding. For example, it remains unknown whether differences in NMJ morphology between species are linked to phylogenetic distance or selective pressure, and we know little about conserved mechanisms within the neuromuscular system of larger mammals. For example, mechanisms contributing to sarcopenia‐related muscle wasting and neurogenic muscle atrophy are primarily being investigated using rodent models, such as a recent study highlighting species‐specific differences and similarities in molecular pathways during muscle ageing between mouse, rat and human (Börsch et al., 2021)—for muscle wasting review see (Tintignac et al., 2015).

**FIGURE 4:**
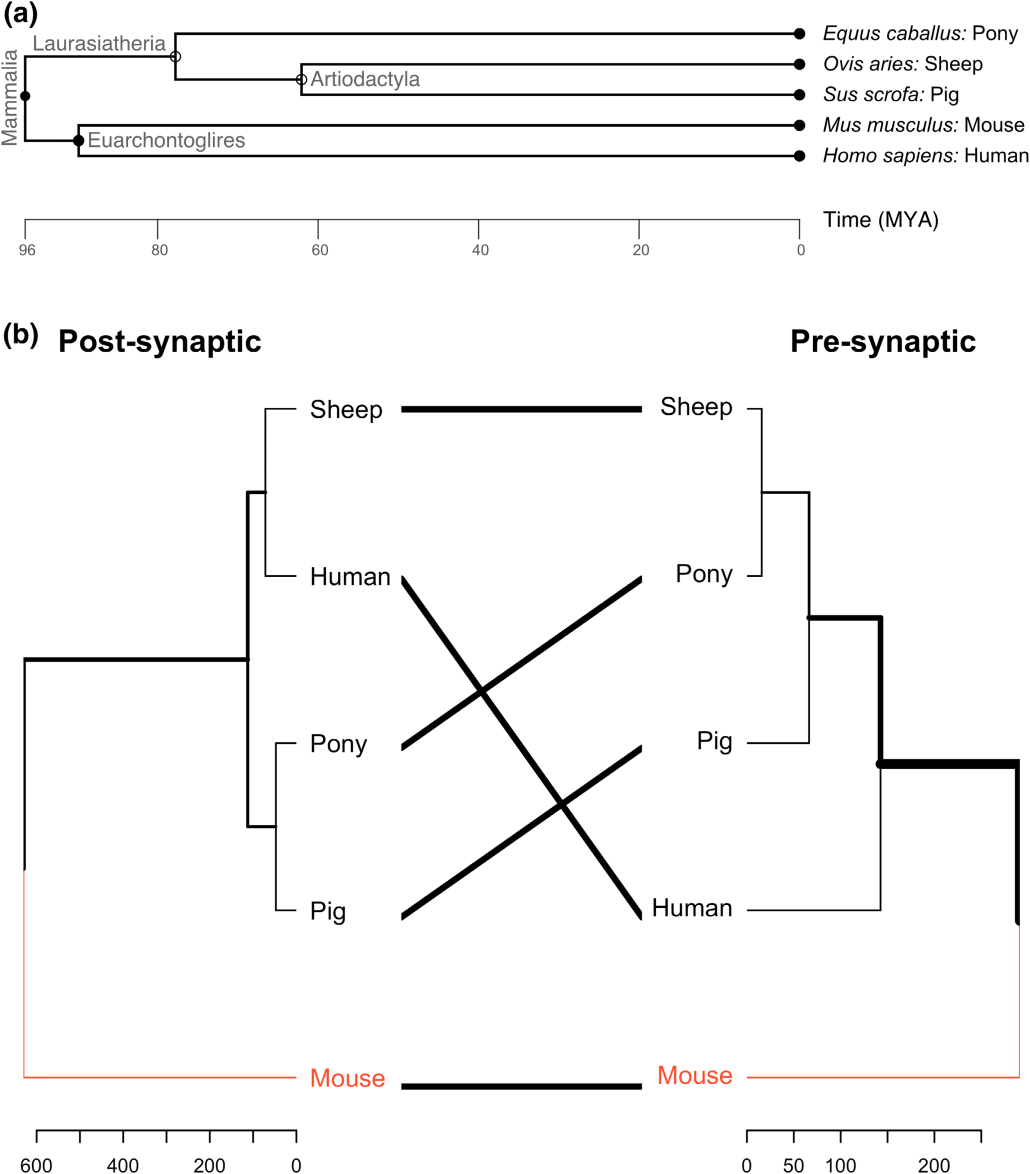
Evolutionary divergence of large mammalian models in comparison to mouse and human. (a) Phylogenetic tree depicting the timeline of divergence in million years ago (MYA) between mouse and human (both part of the superorder Euarchontoglires), sheep and pig (both part of the order Artiodactyla) and pony (all three part of the superorder Laurasiatheria). It is evident that despite mouse and human sharing the same clade, they diverged many million years sooner than the here listed domestic animals. Phylogenetic tree generated on http://www.timetree.org. (b) This so‐called tanglegram showcases the difference between two dendrograms. In this case, the individual dendrograms depict pre‐ and post‐synaptic components of the NMJ. Comparison via such a tanglegram allows to assess the differences or similarities between species, across pre‐synaptic, or post‐synaptic components of the NMJ. Both dendrograms showcase clustering of species according to the similarity in post‐synaptic (left dendrogram) or pre‐synaptic (right dendrogram) NMJ variables and their associated and derived variables resulting from analysis with NMJ‐morph/aNMJ‐morph (Jones et al., 2016; Minty et al., 2020). Red lines indicate similarities in subtree branches: the mouse is most different from the other species in post‐ and pre‐synaptic morphology. Thick black lines at the edges of the dendrogram indicate differences in branch distance from their node of origin: whilst sheep and human are most similar in their post‐synaptic morphology, the sheep and pony are most similar in their pre‐synaptic morphology. Mouse and human data were reproduced from (Jones et al., 2017). Sheep and pig data were reproduced from (Boehm, Alhindi, et al., 2020; Boehm, Miller, et al., 2020). Pony data yet unpublished (Cahalan et al., 2022, under review). Tanglegram was generated in RStudio (version 1.4.0) using the packages tidverse, usedist, vegan, magrittr and dendextend (Galili, 2015).

Since humans and mice are descended from a younger common ancestor (the superorder Euarchontoglires) than the sheep, pig and pony (the superorder Laurasiatheria), which diverged later, one might assume that murine models are more similar to the human (Figure 4(a)). However, phylogenetic divergence does not necessarily inform us about similarity of genetic sequence or morphology. For example out of 22 select genes associated with neuromuscular disorders, the pig has the highest percentage of nucleotide sequence identity to the human as compared with dog, mouse and rat (Barthélémy et al., 2019). Since there are species‐specific differences in pre‐ and post‐synaptic morphology between species (Figure 3), one might wonder whether motor nerve (pre‐synapse) or target skeletal muscle fibre (post‐synapse) underwent different functional adaptations, or whether purely genetic drift was responsible for species‐specific differences. In the case of genetic drift, one would expect both pre‐ and post‐synaptic NMJ morphology of species from Figure 3 to cluster as they do in their phylogenetic tree (Figure 4(a)). Whilst mouse NMJ morphology is strikingly different from those of sheep, pig, human and pony, at both pre‐ and post‐synapse, neither pre‐ nor post‐synaptic NMJ morphology across species matches their phylogenetic divergence (Figure 4(b)). Despite small differences in their clustering between pre‐ and post‐synaptic NMJ morphology, those of sheep and pig, (at least in select pelvic limb muscles), are remarkably similar to those of humans, as exemplified by recent comparative work (Boehm, Alhindi, et al., 2020; Boehm, Miller, et al., 2020).

A study of NMJ morphology between *Drosophila* species showed a similar result: whilst differences in NMJ morphology were found between *Drosophila* species, these were not aligned with phylogenetic distance between these species—differences in *Drosophila* NMJ structure and function result from selection pressure and adaptation to environmental factors rather than purely genetic drift (Campbell & Ganetzky, 2012). Data from dogs suggest a similar conclusion and indicates that larger mammals might be genetically more similar to humans than rodents (Barthélémy et al., 2019; Wang et al., 2013); selection pressure due to environmental factors and functional adaptations might shape both genetic factors and associated NMJ morphology.

Advances in molecular biology and sequencing technologies will allow us to shed light on conserved pathways associated with NMJ morphology and function between larger mammalian models and humans, and might uncover the mechanisms of parallel evolution that can ultimately aid in our translational efforts and drug discovery in certain neuromuscular diseases.

## 6 AN UNEXPLORED AREA OF NMJ RESEARCH: LARGE MAMMALIAN NMDS

Comparatively little is known about healthy large mammalian NMJ morphology in general, and this is condensed within a few recent papers. This knowledge deficit is more conspicuous in the field of large animal neuromuscular diseases, where there is little to no published NMJ data.

As previous sections have outlined, significant differences exist between human and rodent models. For example compared to rodents, it seems reasonable that large animal models, with similar axon lengths to humans, will reveal more about neuropathies with a length‐dependency.

Axonopathies, characterised by axonal degeneration and ultimately fragmentation, are the most common form of PNS disease in all species (Lanigan et al., 2021). The nerve fibres are affected in a length‐dependent pattern in distal dying‐back axonopathies. In humans, height is correlated with an increased risk of various peripheral neuropathies, including in HIV and type 2 diabetes patients (Cheng et al., 2006; Cherry et al., 2009). Thus, taller subjects are more likely to develop lower extremity peripheral neuropathy, with a cut‐off at >1.70 m of height (Cherry et al., 2009). This is likely because the longer the nerve, the more vulnerable the axon is to insult, and to disturbances in axonal transport, likely because of its exaggerated metabolic demands. Therefore, the first advantage of using large mammals is a better recapitulation of the length of affected nerve. For example pigs and sheep have recently successfully been used as preclinical models to study nerve regeneration following peripheral nerve injury (Alvites et al., 2021; Burrell et al., 2020), suggesting potential for future translational clinical applications to humans and other veterinary species.

Given the deficiency of knowledge regarding large mammal NMJ morphology in disease states, it seems reasonable that its study will have translational impact, allowing a better understanding of changes at the human NMJ. As such, what follows is a summary of pertinent large animal NMDs. For each, there is an opportunity for NMD translational discovery.

## 7 HORSES

### 7.1 Equine recurrent laryngeal neuropathy (RLN)

Equine recurrent laryngeal neuropathy (RLN) is a common neuromuscular condition primarily affecting tall horse breeds such as Thoroughbreds and various Draughts (Draper & Piercy, 2018); as a neurodegenerative disorder affecting the recurrent laryngeal nerves (RLn)—the longest equine motor axons, measuring up to 2.5 m—it is likely one of the more prevalent, length‐dependent neuropathies in large mammals. It is characterised by varying degrees of arytenoid cartilage paresis, primarily on the left side, likely because the left‐sided nerve is longer than that on the right side. Indeed, evidence suggests that most, if not all, large breed horses have varying severities of this disorder (Draper & Piercy, 2018). Affected horses produce abnormal respiratory sounds during exercise and show exercise intolerance in the most severe cases caused by the associated paresis of the denervated *cricoarytenoideus dorsalis* muscles that normally abduct the vocal cords, opening the rima glottidis. Despite the high prevalence, the exact cause of RLN remains unclear, though it likely includes acquired and genetic factors (Draper & Piercy, 2018). Length‐dependency is also a common feature of human peripheral neuropathies that have a genetic basis, such as in CMT 1A (Krajewski et al., 2000; Scherer & Wrabetz, 2008), or in certain acquired diabetic neuropathies (Kazamel & Dyck, 2015). Typically, CMT involves the distal extremities, although a few patient subtypes (mainly CMT4A, CMT2A and CMT2C—select familial examples are mentioned in the section ‘Translational pitfalls in rodent NMJ form and function’) also develop laryngeal paralysis (Zambon et al., 2017). Note that in some patient subsets, CMT first presents with atrophy and weakness of the intrinsic muscles of the hands, without involvement of lower limbs until later in the disease course, indicating the clinical heterogeneity of CMT disorders (McMacken et al., 2021) and the presence of disease factors beyond pure length‐dependency. Some of the neuropathological features associated with CMT diseases (particularly CMT2A, E and F), such as loss of myelinated nerve fibres and organelle‐containing paranodal evaginations (Millecamps & Julien, 2013), also occur in RLN (Duncan, 1978)—the study of the equine NMJ in these cases might then yield translatable insights into chronic NMJ (mal)adaptions in these disorders. Recently, novel treatments for RLN have shown promising results. For example a cervical nerve transplantation technique enabled reinnervation of the cricoarytenoideus dorsalis muscle (Rossignol et al., 2018) in affected horses.

### 7.2 Equine motor neuron disease (EMND)

Equine motor neuron disease is a neurodegenerative neuronopathy characterised by generalised paresis, muscle fasciculations, muscle atrophy and progressive weight loss (Banfield et al., 2019; Sasaki et al., 2016; Sisó et al., 2006). Pathology studies show motor neuron degeneration in the spinal cord and brain stem, leading to axonal degeneration in the CNS and PNS. The aetiology appears to be related primarily to an acquired deficiency of anti‐oxidants, especially of vitamin E (Mohammed et al., 2007).

### 7.3 Acquired equine polyneuropathy (AEP)

Acquired equine polyneuropathy is a sometimes‐fatal neurological disease characterised by pelvic limb paresis. It has been mainly described in Sweden, Norway and Finland and is also referred to as ‘Scandinavian knuckling syndrome’ (Gröndahl et al., 2012; Hanche‐Olsen, Kielland, et al., 2017). Despite the geographical pattern and association with forage feeding, the aetiopathogenesis remains unclear. Affected horses present with a polyneuropathy with inflammatory demyelination and Schwann cell inclusions, suggestive of a primary Schwannopathy (Hanche‐Olsen, Kielland, et al., 2017, Hanche‐Olsen, Matiasek, et al., 2017). These horses, regardless of size, develop recurrent laryngeal nerve lesions yet do not demonstrate clinically defective laryngeal function.

## 8 GOATS

Laryngeal neuropathy has been described in goats with clinical signs of copper deficiency (Sousa et al., 2016). The main lesions were axonal degeneration of the RLns and atrophy of intrinsic laryngeal muscles. Another acquired peripheral neuropathy in the goat is caused by coyotillo (*Karwinskia humboldtiana*, also known as Humboldt's Buckthorn) poisoning (Charlton et al., 1971), where the results suggested a primary mitochondrial injury in Schwann cells with resulting impaired axonal transport, myelin splitting and segmental demyelination in long nerves such as the sciatic. A subclinical demyelinating polyneuropathy was recently studied in goats (Skedsmo et al., 2020). This disease was caused by the loss of the cellular prion protein (PrPC), confirming the importance of PrPC for peripheral nerve myelin maintenance.

## 9 SHEEP

The most common neurodegenerative disorder described in sheep is neuroaxonal dystrophy, characterised by numerous axonal swellings, myelin loss and axonal degeneration, particularly in the spinal cord and sciatic nerve (Finnie & Manavis, 2017). It has been observed in juvenile and newborn Australian Merino lambs and Suffolk sheep (Bourke, 1995; Harper et al., 1986; Sisó et al., 2006).

As previously mentioned, the ovine NMJ most closely resembles the human NMJ (Figure 3) (Boehm, Alhindi, et al., 2020; Boehm, Miller, et al., 2020). Sheep have been used as models of peripheral nerve injury affecting the cervical nerve roots (Hems & Glasby, 1992), C6 ventral root avulsion (Fullarton et al., 2001) and the facial nerve (Starritt et al., 2011). Sheep models have been used to recapitulate Batten disease (Weber & Pearce, 2013), and the first gene‐edited ovine model of neuronal ceroid lipofuscinoses has recently been generated (Eaton et al., 2019).

Additionally, aged sheep are used as a model for functional electrical stimulation of the recurrent laryngeal nerve, advancing the understanding and the clinical translation of conditions with atrophied laryngeal muscles such as vocal fold paralysis (Gugatschka et al., 2018; Karbiener et al., 2016).

## 10 PIGS

Similar to the ovine NMJ, porcine NMJs closely resemble those of humans (Figure 3), improving the translational potential of this species for the study of motor neuron diseases (Boehm, Alhindi, et al., 2020, Boehm, Miller, et al., 2020). A spontaneous porcine motor neuron disease (SPMND), with features similar to the equine disorder, has been described in feeder pigs (Wohlsein et al., 2012). A putative peripheral neuropathy with unclear aetiology has been described in suckling piglets (Sályi et al., 2000). This was characterised by degeneration, demyelination and necrosis of the tibial nerve and the common fibular nerve, with no CNS involvement.

Pigs are used to model Spinal Muscular Atrophy (SMA), a human genetic disorder characterised by motor neuron degeneration and paresis (Duque et al., 2015). Results from porcine models and other large animals of SMA have not only shed light on the molecular mechanisms of the disease, they have also provided valuable insights into biomarkers and gene delivery strategies, therefore allowing a quicker advancement of gene therapy and similar molecular approaches to the clinic (Bevan et al., 2011; Iyer et al., 2017).

## 11 CATTLE

CMT type 4H in humans arises through homozygous mutations in the *FGD4* gene. A recent study of Holstein Friesian cattle with a homozygous splice site mutation in this gene revealed clinical signs of stumbling and loss of coordination in animals close to 2 years of age (early adulthood) (Dittmer et al., 2022). Gross post‐mortem abnormalities were not observed. Examination of a range of peripheral nerves revealed demyelination and remyelination, with Schwann cell hyperplasia and hypertrophy, onion bulb formation and decreased myelinated fiber density. These changes can also be found in human CMT type 4H and in *FGD4* KO mouse models (Dittmer et al., 2022).

Bovine spastic paresis (BSP) is a relatively common progressive NMD affecting many breeds of cattle and is characterised by spastic contractions of one or more pelvic limb muscles. The *gastrocnemius* muscle is the most commonly affected, with spastic paresis causing the animal to repetitively stretch the affected limb caudally. BSP likely has a genetic basis, however, the exact aetiopathogenesis remains unknown—histopathology of the spinal cord, tibial nerves and muscle tissue of affected animals do not reveal abnormalities. A functional pathology occurring from overstimulation and/or lack of inhibition from centrally controlled spinal cord γγ motor neurones is postulated (De Vlamynck et al., 2014).

## 12 LLAMAS AND ALPACAS

Paralysis of the diaphragm with phrenic nerve degeneration has been reported in llamas and alpacas (Bedenice et al., 2002; Byers et al., 2011; Uzal et al., 2012). Neuropathological studies showed that affected axons varied from being intact to being vacuolated and degenerated with loss of neurofilaments. The aetiology of this phrenic nerve neuropathy could not be elucidated.

## 13 THE PROMISE AND PRACTICE OF STEM CELL WORK

Stem cell technologies have emerged over the last two decades to create a field with much promise for generating therapeutics and cellular regenerative biology insights for chronic degenerative disorders (Zakrzewski et al., 2019). Insights into the physiological and pathological function of the NMJ might come from iPSC‐derived models (Lin et al., 2019; Thompson et al., 2012), or cultured neurons, which have now been generated from large mammals (Bressan et al., 2020; Pessôa et al., 2019), including horses (Adalbert et al., 2022). iPSCs can be differentiated into muscle or neural tissue, with a future promise of in vitro NMJ models (Jongh et al., 2021), providing an understanding of the cellular and molecular mechanism and the aetiology underlying many NMJ‐related disorders and peripheral neuropathies. Besides offering a possible disease modelling platform, iPSC‐ and other cell‐based models can also act as an ex vivo platform to test potential therapeutic strategies and drugs.

Large animal models are essential for the translation of therapeutics that utilise stem cell and tissue engineering strategies (Ribitsch et al., 2020). In addition, trials to treat large animals (e.g., dogs and horses) with stem cell‐ and biomaterial‐based therapies are also underway. For example, stem cell therapy using adult mesenchymal stem cells derived from bone marrow is approved in equine medicine for musculoskeletal disorders (Ortved, 2018). Veterinary regenerative medicine is growing in popularity (Barrachina et al., 2018; Koch et al., 2009; Smith et al., 2014). In the future, these novel approaches could be applied to peripheral nerve regeneration in humans, providing a treatment for peripheral neuropathies.

## 14 WEIGHING THE TRANSLATIONAL BENEFITS AND COSTS OF STUDYING NMJS IN LARGE ANIMAL MODELS

The complexity of human diseases necessarily means that no one animal model will likely replicate all aspects of the disease. However, to facilitate the most efficient translation from bench to bedside, the research community should aim to recapitulate the condition wherever possible (Eaton & Wishart, 2017). Murine models are currently the most popular model for the study of human disease–in particular due to their quick reproductive rate, low maintenance cost, ease of genetic manipulation and variety of experimental tools developed to study them (Chung et al., 1997)–The emergence of nuclease‐mediated genome editing technology (CRISPR/Cas9) however, recently used to create a knock‐in pig model with features of Huntingdon's disease (Yan et al., 2018), has greatly improved the efficiency of generating genetically modified animals—see review of genetically modified neurogenerative large animal models (Yang et al., 2021). Thus, the appeal of large animal models across a range of clinical applications should be considered.

Despite the comparatively higher cost and level of maintenance and workforce involved with large animals, the benefits that the similarity of these models could bring, should be considered as an encouragement for the research community. A higher cost is somewhat offset by the very high prevalence of certain large animal diseases–for example RLN has a cited worldwide clinically relevant occurrence of between 2 and 11% in Thoroughbred horses (Boyko et al., 2014), whereas most human neuropathies are comparatively rare. A high natural prevalence of certain large animal diseases might negate the need to maintain colonies of affected animals, with associated welfare and ethical advantages.

Neurodegenerative conditions that occur naturally in large animals and humans, such as the neuropathies outlined above, should be of particular benefit for clinical translation (Eaton & Wishart, 2017), as one could expect more commonalities in disease onset and progression.

More translatable data ultimately contributes to a reduced failure rate of therapeutics within the drug discovery pipeline, as currently, which still occurs commonly in human clinical trials (Seyhan, 2019). Given that drugs typically take over 12 years to get from the lab through to approval and development costs can exceed $1 billion (Mohs & Greig, 2017), it is in everyone's interest to accelerate this process and reduce the attrition rate of therapeutics and also reduce associated costs. Animal models that better mimic human NMJ morphology, and length dependency of axon functions, will hopefully allow researchers to identify drugs that are less likely to fail in clinical trials, whilst reducing costs.

## 15 CONCLUSIONS

The species and disease model of choice are undoubtedly relevant to answering both research questions and clinical problems. The aetiology of peripheral neuropathies in large animals is often undetermined, and NMJ involvement is overlooked. Large animal models have great potential to enhance our understanding of the neuromuscular system in health and disease. Although elevated costs can constrain large animal studies, their high prevalence and application of a more appropriate comparative approach should help close the translational gap between preclinical and clinical responses.

## FUNDING INFORMATION

Horserace Betting Levy Board (HBLB) Grant & Anatomical Society Prize PhD Studentship.

### DATA AVAILIBILITY STATEMENT

The data that support the findings within this review are openly available at https://github.com/Boehmin/NMJ_analysis.git
